# Interactions between flue gas desulfurization gypsum and biochar on water infiltration characteristics and physicochemical properties of saline-alkaline soil

**DOI:** 10.1007/s10661-023-11894-3

**Published:** 2023-10-06

**Authors:** Peijun Wang, Xiaolan Lin, Qi Liu, Ziqi Lin, Yali Yang, Hui Chen, Shenglong Fan

**Affiliations:** 1 Research Center for Land Use and Ecological Security Governance in Mining Area, School of Public Policy and Management, China University of Mining and Technology, Xuzhou, China; 2 College of Resources and Environment, Fujian Agriculture and Forestry University, Fuzhou, China; 3 School of Public Administration and Law, Fujian Agriculture and Forestry University, Fuzhou, China; 4 Natural Resources Service Center, Pingtan Comprehensive Environmental Zone, Fuzhou, China

**Keywords:** Flue gas desulfurization gypsum, Biochar, Infiltration, Physicochemical properties, Saline-alkali soil

## Abstract

The application of flue gas desulfurization gypsum (FGDG) improves the soil structure, reduces soil pH, and accelerates soil salt leaching. Biochar amendment to soil can affect the soil infiltration rate, increase soil porosity, decrease soil bulk density, and enhance the water retention capacity. This study investigated the interactive effect of FGDG and biochar on water infiltration characteristics and physicochemical properties as well as determined the optimal amendment rate as a saline-alkaline soil conditioner. Seven experimental schemes were designed, and the newly reclaimed cultivated soil from Pingtan Comprehensive Experimental Zone in Fujian Province, China, was used in an indoor soil column experiment to simulate soil infiltration. Five models were employed to describe the infiltration process. The power function was used to represent the dynamic process of the wetting front. The conclusions of this study are as follows: (1) there was a reduction in the infiltration capacity of saline-alkaline soil (sandy soil) in each treatment, and the application of FGDG alone had the highest inhibition effect compared to the control (CK). The Kostiakov model provides the best fit for the experimental data of soil cumulative infiltration. (2) All treatments increased the total porosity and water content of saline-alkali soil, with the combined application of FGDG and biochar found to be more effective. (3) The application of FGDG alone or in combination with biochar decreased the pH and increased the electrical conductivity of the saline-alkali soil significantly, with the combined application having the most significant effect. In contrast, soil amended with biochar alone had minimal effect on the pH and EC of the soil. (4) The best improvement ratio was achieved with the F1B2 combination (75 g/kg FGDG + 30 g/kg biochar).

## Introduction

The salinization of soil is one of the major global problems affecting grain productivity and the quality of the environment (Singh, 2016). Around one billion hectares of saline-alkaline soil can be located in more than 100 countries (Liu et al., 2018; Qadir et al., 2006; Wong et al., 2010; Xia et al., 2019). The saline-alkali land in China is widely distributed and primarily located in the inland regions of Northwest, Northeast, and North China as well as the semiarid regions of the middle and upper reaches of the Yellow River and coastal regions (Liu et al., 2019), covering a total area of approximately 3.6 × 10^7^ ha (Li et al., 2021a). The coastal saline-alkali land accounts for approximately 2.17 × 10^6^ ha (Long et al., 2016; Zhang et al., 2020a, b) and is a critical land resource with considerable potential for development (Bai et al., 2018; Zhang et al., 2019, 2020a, b). The rapid economic development in the coastal regions has led to increased land demand. Using abundant tidal flat reserves for land reclamation can effectively bridge the gap between a large population and the availability of limited land resources in coastal regions (Saifullah et al., 2018; Zhang et al., 2020a, b). However, the new farmlands developed on the reclaimed coastal tidal flats experience deterioration in soil physical characteristics, such as high bulk density, low hydraulic conductivity, loss of soil nutrients, inhibition of seed germination, and decline in crop yields (Ganjegunte et al., 2014; Li et al., 2021b; Luo et al., 2017; Mao et al., 2016). Several strategies have been implemented to improve the nutrient content and agricultural yield of saline-alkaline soil, for example, irrigation (Heng et al., 2018), tillage methods (Strudley et al., 2008; Singh et al., 2016), the addition of sediments (Li et al., 2022; Mao et al., 2016, 2018), incorporation of plant residues (Li et al., 2017; Zhu et al., 2021), chemical remediation (Ahmad et al., 2013; Gharaibeh et al., 2010; Kharel et al., 2018; Xiao et al., 2022), organic amendments (Rezapour et al., 2022; Yang et al., 2018), and plantation of salt-resistant species (Li et al., 2019).

In recent years, the effectiveness of biochar as a soil amendment has been widely recognized (Abbas et al., 2022; Sadegh-Zadeh et al., 2018; Wang et al., 2022). It is characterized by high porosity, carbon-rich composition, and fine granular structure and is produced through pyrolysis of biomass at high temperatures and low oxygen levels (Cui et al., 2021; Kamali et al., 2022). The increasing scientific evidence has revealed that biochar amendment to the soil leads to a significant decrease in the salinity and pH of saline-alkaline soils, while simultaneously improving the cation exchange capacity (CEC), soil organic matter (SOM), nutrient bioavailability, and plant growth in the soil (Abbas et al., 2022; El-Naggar et al., 2019; Luo et al., 2017; Saifullah et al., 2018; Ullah et al., 2020; Wang et al., 2022). On the other hand, improper application of biochar to saline-alkaline soil may lead to minimal or no effect on soil fertility and plant growth (Mukherjee & Lal, 2014). In addition, the impact of biochar on saline-alkaline soil depends on the type of soil and biochar as well as the amount of biochar (Saifullah et al., 2018). There is an increased demand for biochar suitable for the reclamation of saline-alkali soil.

Flue gas desulfurization gypsum (FGDG) is a byproduct of sulfur (S) removal from fuel combustion gases in coal-based power generation plants (Wang & Yang, 2018). CaSO_4_ is the major component of FGDG, although it may also contain other trace elements (Koralegedara et al., 2019). It is now widely accepted that the application of FGDG to saline-alkali soils can provide a high concentration of Ca^2+^ ions that replace Na^+^ ions on soil colloids. This can enhance soil fertility and crop yield as well as improve the soil structure, decrease soil pH, and reduce salinity levels (Morsy et al., 2022; Murtaza et al., 2017; Qadir et al., 2022; Temiz & Cayci, 2018; Zhang et al., 2020a, b; Zhao et al., 2018; Zhu et al., 2020). However, the quantity, timing, and mode of FGDG application can vary depending on the soil type, the type of crop variety, the prevailing agro-climatic conditions, and the type of available FGDG (Wang & Yang, 2018).

The extensive application of FGDG and biochar for soil improvement is substantiated by the results of several studies conducted on the single or combined application of FGDG and biochar (Zhang et al., 2017, 2020a, b; Zhu et al., 2020). For example, Zhang et al. (2017) demonstrated that the application of FGDG in combination with biochar was more effective than only FGDG. Zhang et al., (2020a, b) reported that the co-application of gypsum and biochar could increase the hydraulic conductivity of saline-alkaline soil and minimize sodicity caused by short-term leaching. The combined application of FGDG and biochar has yielded positive results; however, there are certain shortcomings as follows: firstly, the effect of the combined application of FGDG and biochar on the improvement of saline-alkali sandy soil has not been reported to date. Secondly, the improvement strategy reported in previous studies was relatively simple. The researchers only compared the single and combined applications, with the combination of FGDG and biochar applied only in the ratio of 1:1. There is a lack of research on the interplay of integrated applications. The majority of studies have focused on the effect of improvement measures on soil salinity and alkalinity, whereas relatively few studies have examined its impact on soil infiltration properties and water-holding capacity. Infiltration plays a vital role in the terrestrial water cycle, transforming surface water into soil and groundwater (Wang et al., 2017a). During irrigation events, infiltration is a crucial dynamic process that must be considered for designing, scheduling, optimizing, and management of irrigation systems (Duan et al., 2011).

To bridge the research gap, the following hypotheses were proposed in this study: (1) to evaluate the suitability of Philip, Kostiakov, Horton, Mezencev, and USDA-NRCS models for the comparison of the soil infiltration characteristics of saline-alkali soil under varying FGDG and biochar application rates; (2) the co-application of FGDG and biochar could improve the soil water content when compared to only biochar due to the ameliorative effect on the total porosity of soil; and (3) the interaction between FGDG and biochar addition on reduction of soil pH is more significant. The study aimed to test these hypotheses by conducting indoor soil column-based infiltration experiments by applying four different levels of FGDG and biochar as well as without FGDG or biochar. The goal of this study was to demonstrate the effect of FGDG and biochar on the infiltration properties, soil water content, total porosity, pH, and electrical conductivity (EC) of saline-alkali soil (sandy soil).

## Materials and methods

### Test materials

The experimental site selected for this study was newly reclaimed tidal land located in the Pingtan Comprehensive Experimental Zone in Fujian Province, China. The soil sampling depth was 60 cm (0–20 cm surface soil; 20–60 cm subsoil). Impurities in the soil sample were removed, and then, samples were air-dried and passed through a 2-mm sieve to determine the physicochemical properties. The results of the analysis are presented in Table 1. The collected soil sample represents saline-alkali soil type and has a sandy texture. The particle size of FGDG corresponded to 0.048–0.074 mm, the content of CaSO_4_ was approximately 40%, pH was 6.9, and water content was about 0.5–1%. Biochar was produced in a high-temperature pyrolysis carbonization furnace at 750 °C (bamboo biochar was characterized by a particle size of 0.5–3 mm, moisture content of 8.26%, pH of 8.5, and organic carbon content of 78.12 g/kg).

**Table 1:** Basic physicochemical properties of saline–alkali soil

Soil properties	Units	Soil depth	
		0–20 cm	20–60 cm
Particle size distribution	< 0.002 mm (%)	1	1
	0.002–0.02 mm (%)	5.49	6.5
	> 0.02 mm (%)	93.51	92.5
Soil texture^a^		Sandy soil	Sandy soil
Soil bulk density	g·cm^−3^	1.6	1.5
Soil electrical conductivity	ms·cm^−1^	0.053	0.056
pH		8.9	8.7
Organic matter	g·kg^−1^	2.079	10.428
Total nitrogen	g·kg^−1^	0.063	0.086
Hydrolyzable nitrogen	mg·kg^−1^	0.610	1.990
Total phosphorus	g·kg^−1^	0.098	0.002
Available phosphorus	mg·kg^−1^	16.580	15.050
Total potassium	g·kg^−1^	20.458	20.458
Available potassium	mg·kg^−1^	182.767	162.661
Water soluble sodium ion	g·kg^−1^	0.079	0.098
Water soluble potassium ion	g·kg^−1^	0.091	0.114
Water soluble calcium ion	g·kg^−1^	0.321	0.527
Water soluble magnesium ion	g·kg^−1^	0.067	0.061
Carbonate ion	g·kg^−1^	0.061	0.065
Bicarbonate ion	g·kg^−1^	1.514	1.207
Water soluble chloride ion	g·kg^−1^	0.053	0.040
Sulfate ion	g·kg^−1^	2.476	2.070
Exchangeable sodium ion	g·kg^−1^	0.040	0.044
CEC	cmol·kg^−1^	4.020	4.709

^a^According to the international soil texture classification system

## Experimental design and methodology

### Experimental scheme

From May 5 to July 10, 2021, soil column-based infiltration experiments were conducted in the College of Resources and Environment lab at Fujian Agriculture and Forestry University. In these experiments, the bottom layer was composed of only sandy soil (20–60 cm), whereas the surface layer was composed of soil combined with FGDG and biochar (0–20 cm). Table 2 shows the ratios of addition for both single and combined applications. Each experiment was performed in triplicate, with a total of seven treatments.

**Table 2:** Experimental schemes of various treatments with single and combined application of FGDG and biochar

Soil layer	Experimental treatments						
	1	2	3	4	5	6	7
0–20 cm	CK No amendments	F^a^ F 100 g/kg soil	B^b^ B 50 g/kg soil	F1B1 F 75 g/kg soil B 20 g/kg soil	F1B2 F 75 g/kg soil B 30 g/kg soil	F2B1 F 50 g/kg soil B 20 g/kg soil	F2B2 F 50 g/kg soil B 30 g/kg soil
20–60 cm	Sandy soil	Sandy soil	Sandy soil	Sandy soil	Sandy soil	Sandy soil	Sandy soil

^a^F stands for FGDG

^b^B stands for biochar

### Design of infiltration experiment apparatus

**Fig. 1:**
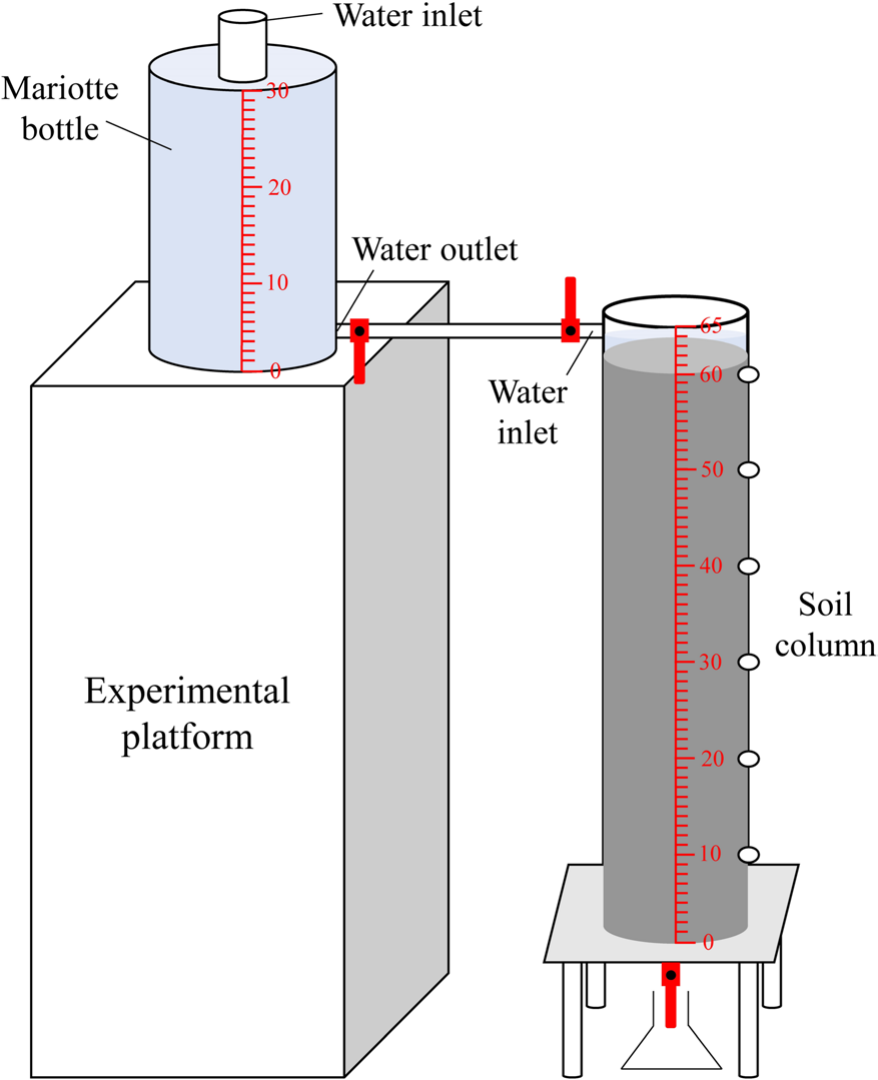
Indoor infiltration experimental apparatus

A transparent acrylic tube with a 10 cm diameter and 65 cm height was used as the soil column apparatus in the infiltration experiment. Based on the principle of 10 cm per layer, seven small holes (*d* = 2 cm) were made on the transparent acrylic tube for soil sampling, and a precise scale was engraved on the tube for direct reading. The water supply system of the infiltration apparatus comprised a Mariotte bottle with an inner diameter of 12 cm and a height of 38 cm. The outer wall was calibrated for the measurement of the water level. A small hose was provided to connect the soil column and Mariotte bottle, and a small valve controlled the water flow in and out of the tube. The infiltration apparatus set up for the experiment is shown in Fig. 1. Before soil loading, a 100-mesh nylon sieve and a piece of filter paper (*d* = 10 cm) were placed at the base of the column to prevent the loss of soil particles. In addition, a layer of Vaseline was applied on the inner wall of the transparent acrylic tube to minimize the marginal effect in the infiltration process. Based on the bulk density of the tested soil, each 10-cm layer of soil was added to the soil column, and the interspace between layers was compacted. The soil column had a total height of 60 cm. The columns in the control (CK) group were packed with sandy soil only. For the remaining six treatments, the specified amount of amendment and soil were evenly mixed in the surface layer (0–20 cm), and the bottom layer (20–60 cm) was packed with sandy soil only.

### Determination method

The infiltration characteristics of saline-alkali soil (sandy soil) were measured using a one-dimensional vertical stagnant infiltration method with a constant water head. The position of the Mariotte bottle was adjusted to maintain the water head at approximately 4 cm, and the outlet valve of the Mariotte bottle was kept open. Once the outlet hose had been filled, water was added up to a height of 4 cm above the surface of the soil column, and an infiltration experiment was conducted to observe the decline in water level in the Mariotte bottle as well as the distance traveled by the wetting fronts in the soil columns. The falling height and the migration distance were recorded every 1 min for the first 30 min, every 5 min for the following 30 to 90 min, every 10 min for the following 90 to 150 min, every 30 min for the following 150 to 300 min, and then every 60 min till the completion of the experiment. The infiltration experiment was concluded as soon as the wetting front reached the bottom of the soil columns.

The soil samples were collected from the sampling holes of the soil columns (one layer per 20 cm; four layers of soil in total) approximately 24 h after the completion of the experiment. After passing through a 2-mm sieve, the soil samples were oven-dried at 105 °C to estimate the soil water content (Hu et al., 2020). The soil–water (1:5 ratio) extracts were prepared to measure the soil pH and EC using a pH meter and conductivity meter, respectively (Qu et al., 2021).

### Fitting of cumulative infiltration in the soil column

The commonly used models such as the Philip model (Philip, 1957), Kostiakov model (Kostiakov, 1932), Horton model (Horton, 1941), Mezencev model (Mezencev, 1948), and USDA-NRCS model (USDA-NRCS, 1974) were fitted to the water infiltration data.

The Philip model can be described using Eq. (1):

1 $$I(t) = St^{0.5} + At$$

where *I*(*t*) represents the cumulative infiltration (cm),* t* is infiltration time (min), *S* is the sorptivity (cm/min^0.5^), and *A* is the saturated hydraulic conductivity (cm/min) (Dashtaki et al., 2009).

The expression for the Kostiakov model is given by Eq. (2):

2 $$I(t) = Kt^{n}$$

where *I*(*t*) represents the cumulative infiltration (cm), and *K* > 0 and 0 < *n* < 1 are the dimensionless empirical constants (Dashtaki et al., 2009; Wang et al., 2017a).

The Horton model is expressed as follows:

3 $$I(t) = at + \frac{1}{c}(b - a)(1 - e^{ - ct} )$$

where *I*(*t*) represents the cumulative infiltration (cm); *a* and *b* correspond to the steady and initial infiltration rates, respectively; and *c* represents the undetermined parameter.

The Mezencev model can be described using Eq. (4):

4 $$I\left(t\right)={K\mathrm{^{\prime}}}^{b\mathrm{^{\prime}}}+A\mathrm{^{\prime}}t$$

where *I*(*t*) represents the cumulative infiltration (cm), *K′* > 0 and 0 < *b′* < 1 are the dimensionless empirical constants, and *A′* > 0 is the final infiltration rate at the steady-state condition (Wang et al., 2017a).

The USDA-NRCS model is expressed using Eq. (5):

5 $$I\left(t\right)={d\mathrm{^{\prime}}t}^{b\mathrm{^{\prime}}\mathrm{^{\prime}}}+0.6985$$

where *I*(*t*) represents cumulative infiltration (cm), and *a″* and *b″* are dimensionless empirical constants (Duan et al., 2011; Wang et al., 2017a).

### Fitting of wetting front migration of soil column

A power function was employed to simulate the collected data on the correlation between the migration distance of wetting front *D* (cm) and the infiltration time *t*.

6 $$D = at^{b}$$

where *a* represents the wetting front migration distance within the first unit of time, and* b* represents the attenuation of the wetting front advance process (Li et al., 1999).

### Model evaluation and data processing

Excel 2021 (Microsoft Corporation, Redmond, WA, USA) was utilized for sorting the initial data and estimation of the average of triplicates. OriginPro 2022 (OriginLab, Northampton, Massachusetts, USA) was used for data fitting and plotting. The differences between the treatments were analyzed using the one-way ANOVA method with IBM SPSS v. 26.0 (IBM Corp., Armonk, NY, USA), and the Duncan method was used for multiple comparisons.

All five models of soil infiltration were non-linear. Therefore, the models were assessed using the reduced Chi-Sqr values and the adjusted coefficient of determination (Adj-*R*^2^) (Wang et al., 2017a). The values of Adj-*R*^2^ close to 1 and reduced Chi-Sqr close to or equal to 0 indicate a greater degree of curve fitting between the independent and dependent variables (Van de Genachte et al., 1996).

7 $$Adj\text{-}{R}^{2}: {}_{R}^{-2}=1-\frac{RSS/dfE\mathrm{^{\prime}}}{TSS/dfE}$$

8 $$\mathrm{Reduced\;Chi\text{-}Sqr:reduced}\quad {x}^{2}=\frac{RSS}{dfE\mathrm{^{\prime}}}$$

where RSS represents the residual sum of squares, TSS indicates the total sum of squares, and dfE′ and dfE represent the error degrees of freedom for RSS and TSS, respectively.

## Results and discussion

### Effect of various experimental treatments on cumulative infiltration

**Fig. 2:**
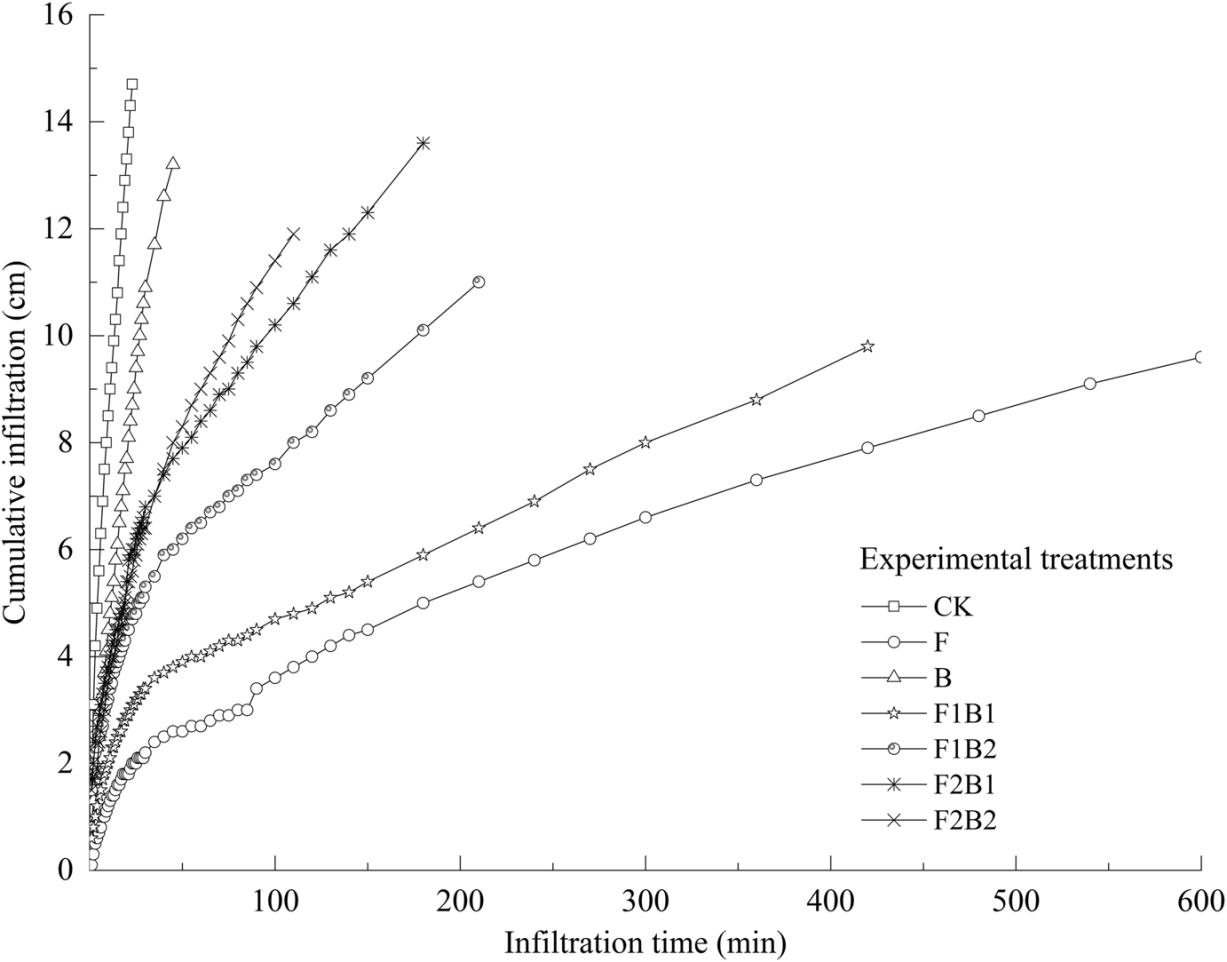
Effects of various experimental treatments on cumulative infiltration; *I* represents cumulative infiltration, and *t* represents infiltration time

Once soil infiltration reaches a steady state, the stable infiltration rate can be used to determine the soil infiltration capacity. On the other hand, cumulative infiltration can be used as a measure of soil infiltration capacity before stabilization. As shown in Fig. 2, all treatments with amendments inhibited the infiltration process, and the degree of inhibition varied among treatments. The time taken for the water to reach the base of the soil column was the shortest for CK, and the infiltration time in the other six treatments was longer than the infiltration time in CK. The infiltration time for different treatments followed the order F (600 min) > F1B1 (420 min) > F1B2 (210 min) > F2B1 (180 min) > F2B2 (110 min) > B (45 min) > CK (23 min). The infiltration time in F treatment was 26.1 times higher than that in CK. The amount of cumulative infiltration for the treatments with amendments was significantly less than that of CK, with a decrease of 34.7% (F), 10.2% (B), 33.3% (F1B1), 25.2% (F1B2), 7.5% (F2B1), and 19% (F2B2). The F treatment exhibited the lowest cumulative infiltration and the most extended infiltration duration, indicating that the F treatment exhibited the most inadequate infiltration capacity. In terms of infiltration time and cumulative infiltration, the infiltration capacity of four combined treatments exhibited the following pattern: F1B1 < F2B1 and F1B2 < F2B2, suggesting an increase in the infiltration time with an increase in the amount of FGDG and a decrease in the amount of cumulative infiltration. In addition, the B treatment had a prolonged period of infiltration compared to CK. Further, although insignificant, the B treatment resulted in a reduction in the cumulative infiltration, indicating a deceleration in the infiltration rate.

The application of FGDG and biochar to saline-alkali soil (sandy soil) delayed infiltration, increased infiltration time, and decreased cumulative infiltration and infiltration capacity. These results contradict the findings of previous studies (Goncalo et al., 2020; Zhang et al., 2020a, b; Bahceci et al., 2022). Zhang et al., (2020a, b) demonstrated that the saturated water content and field water capacity of the biochar treatment significantly increased by 4.4% and 5.6%, respectively, after a short incubation period, and the combined application of gypsum and biochar significantly increased the soil-saturated hydraulic conductivity by 58.4% compared to the single application of either material. Sand accounted for 93.51% of the soil used in this experiment. This sand has coarse particles, a large proportion of ineffective pores, a high infiltration rate, and severe water loss. In contrast, the texture of the saline-alkali soil was silt loam in Zhang et al. (2020a, b). Thus, the major difference between the findings of this study and those of Goncalo et al. (2020), Zhang et al. (2020a, b), and Bahceci et al. (2022) can be attributed to the variations in the soil type and texture, as well as the amounts and ratio of amendments. The effect of amendments on the soil structures with different textures varied significantly, with the types and amounts of amendments having a significant influence on the experimental results (Koralegedara et al., 2019; Saifullah et al., 2018; Singh et al., 2022).

A comparison of four combined treatments (F1B1, F1B2, F2B1, and F2B2) revealed that the increase in the proportion of FGDG with the same amount of biochar increased the degree of inhibition of soil infiltration (*P* < 0.05). The treatments with FGDG application indicated a decrease in the infiltration capacity of soil with an increase in the amount of FGDG compared to CK. The treatment with 10% FGDG alone exhibited the most inadequate infiltration capacity, probably due to the excessive application of FGDG. CaSO_4_ is a salt with medium solubility. As the amount increases, the dissolution of additional solid particles is hindered, which can lead to clogging of soil pores and impede the infiltration process (Al-Kayssi & Mustafa, 2016). The biochar amendment had no significant impact on the infiltration process at the same amount of FGDG (*P* > 0.05). This may be attributed to the significant influence of the amount of FGDG applied rather than biochar applied to soil (Sun et al., 2018) in the composite treatment. Thus, FGDG played a dominant role in the control of the infiltration process.

This study indicated that the application of FGDG and biochar to sandy soil with a coarse texture can reduce water infiltration and hold water in the root zone for an extended period, enabling plants to access more water and nutrients from the soil.

### Effects of various experimental treatments on wetting front migration

**Fig. 3:**
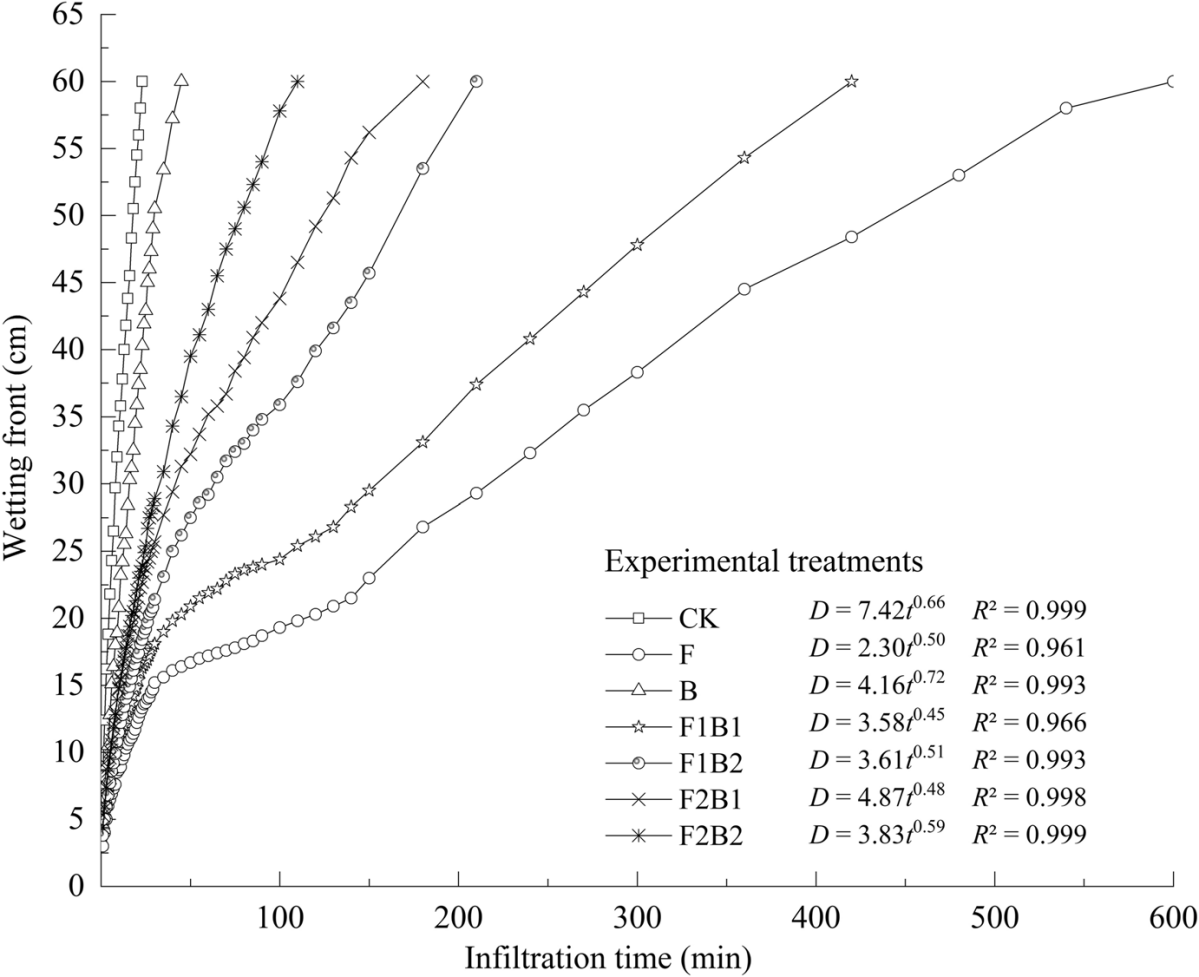
Characteristics of soil wetting front movement under various experimental treatments. *D* stands for wetting front migration distance, and *t* stands for infiltration time

The apparent boundary between the front of the wetting zone and the dry soil layer is referred to as the wetting front, which is influenced by gravity and matrix forces. As shown in Fig. 3, before the displacement of the wetting front to 20 cm, the infiltration rate of each treatment initially increased and then decreased. The soil was initially dry characterized by low water content and high sorptivity. As the infiltration process progresses, the infiltration rate tends to decrease and the difference in the movement of the wetting front between treatments is reduced. Once the migration distance of the wetting front exceeded 20 cm, the slope of the curve and infiltration rate increased, and the difference in the movement of the wetting front between treatments became evident. The migration distances of the wetting fronts at *t* = 23 min corresponded to 60 cm (CK), 13.3 cm (F), 40.3 cm (B), 16.3 cm (F1B1), 18.4 cm (F1B2), 22.7 cm (F2B1), and 23.9 cm (F2B2). The wetting front distance was decreased by 77.8% (F), 32.8% (B), 72.8% (F1B1), 69.3% (F1B2), 62.2% (F2B1), and 60.2% (F2B2). Overall, dynamic change in soil wetting fronts and cumulative infiltration exhibited similar trends in all treatments. CK had the longest wetting front migration distance and rapid water movement, whereas the F treatment had the shortest distance and slow movement of water.

Figure 3 shows that the coefficient of determination of the wetting front was higher than 0.96 for all seven treatments, indicating a good fit and the power regression was appropriate for the dynamic change of the wetting front during infiltration. The curves of F2B2 and CK treatments had the best fit of data. In comparison to the CK, the coefficient *a* for other treatments decreased significantly (*P* < 0.05), and the F treatment had the lowest value. Therefore, the single application of FGDG or the combination of FGDG and biochar significantly shortened the migration distance of the wetting front after the first unit of time and thereby decreased the initial infiltration rate of the soil. Treatment with FGDG alone had the most significant delay, which is consistent with the results of F treatment which had the longest infiltration time. Except for the B treatment, the values of coefficient* b* of other treatments were lower than those of coefficient *b* of CK, indicating that rapid attenuation of the wetting front reduces the infiltration rate in the later stage of infiltration. The coefficient *b* for the B treatment was slightly higher compared to the CK treatment, which demonstrated that the attenuation of the wetting front of the B treatment was slower than that of CK in the later stage of infiltration. Still, the difference was not significant (*P* > 0.05).

In this study, biochar amendment alone led to a decrease in the wetting front migration distance. These results are consistent with the previous findings on sandy soils (Brockhoff et al., 2010; Githinji, 2014; Ibrahim et al., 2017), which may be attributed to the biochar settling in the soil pores, thereby clogging the soil pores and decreasing the water infiltration rates (Ibrahim et al., 2017). In addition, biochar in the soil has the potential to act as a binder, which holds soil particles together and thus restricts the movement of water through the soil pores (Ibrahim et al., 2013). The high porosity and surface area of biochar may also contribute to the decreased water infiltration into sandy soil due to its amendment (Mukherjee & Lal, 2013).

### Model fitting of soil cumulative infiltration of different experimental treatments

The results of the fitting parameters of the five models employed to simulate the soil cumulative infiltration are presented in Tables 3 and 4. The Philip model’s coefficient of determination for all treatments was higher than 0.962. There was a decrease in the parameters *S* and *A* of each treatment compared to CK. Further, there was also a decrease in the sorptivity and steady infiltration rate, indicating the inhibition of the infiltration process by the amendments. However, parameter *A* had negative values in contrast to the actual values. Thus, the Philip model was not found to be suitable for this study. The simulation results for the Horton model demonstrated negative values for *a*, whereas for the Mezencev model, negative values were observed for *A′*. This implied that both models were unsuitable for this study.

**Table 3:** Fitting results of five infiltration models for various experimental treatments with single and combined application of FGDG and biochar

Experimental treatments	Philip model		Kostiakov model		Horton model			Mezencev model			USDA-NRCS model	
	*S* (cm·min^−0.5^)	*A* (cm·min^−1^)	*K*	*n*	*a* (cm·min^−1^)	*b* (cm·min^−1^)	*c*	*K′*	*b′*	*A′* (cm·min^−1^)	*a″*	*b″*
CK	1.933	0.234	1.951	0.640	0.477	1.995	0.403	1.918	0.586	0.111	1.539	0.702
F	0.361	0.001	0.352	0.514	0.013	0.144	0.057	0.436	0.435	0.004	0.153	0.638
B	0.844	0.193	0.824	0.747	− 0.211	0.494	0.015	20.772	0.995	− 20.079	0.576	0.831
F1B1	0.596	− 0.008	0.811	0.396	0.016	0.313	0.098	1.021	0.305	0.007	0.463	0.480
F1B2	1.079	− 0.027	1.427	0.372	0.033	0.563	0.122	1.485	0.353	0.004	1.008	0.427
F2B1	1.316	− 0.027	1.564	0.412	0.047	0.593	0.100	1.506	0.431	− 0.006	1.174	0.459
F2B2	1.158	− 0.092	1.142	0.503	0.068	0.493	0.089	1.001	0.613	− 0.054	0.808	0.566

**Table 4:** Reduced Chi-Sqr and Adj-*R*^2^ values of the five evaluated soil infiltration models for various experimental treatments with single and combined application of FGDG and biochar

Experimental treatments	Philip model		Kostiakov model		Horton model		Mezencev model		USDA-NRCS model	
	Chi-Sqr	Adj-*R*^2^	Chi-Sqr	Adj-*R*^2^	Chi-Sqr	Adj-*R*^2^	Chi-Sqr	Adj-*R*^2^	Chi-Sqr	Adj-*R*^2^
CK	0.034	0.998	0.032	0.998	0.007	0.999	0.032	0.998	0.042	0.997
F	0.026	0.995	0.029	0.994	0.040	0.992	0.022	0.996	0.047	0.991
B	0.110	0.990	0.072	0.994	0.037	0.997	0.050	0.996	0.119	0.990
F1B1	0.150	0.960	0.086	0.977	0.008	0.998	0.064	0.983	0.087	0.977
F1B2	0.082	0.983	0.028	0.994	0.087	0.982	0.028	0.994	0.033	0.993
F2B1	0.062	0.993	0.050	0.994	0.121	0.986	0.050	0.994	0.065	0.993
F2B2	0.028	0.997	0.028	0.997	0.070	0.991	0.017	0.998	0.051	0.994

The estimates of Chi-Sqr and Adj-*R*^2^ indicated that the fit of the Kostiakov model was better than that of the USDA-NRCS model. The Kostiakov model was more appropriate for fitting the experimental data in this study. The parameter* K* in the Kostiakov model represents the cumulative infiltration within the first unit of time and characterizes initial infiltration capacity. The value of *K* decreased by 82% (F), 57.8% (B), 58.4% (F1B1), 26.9% (F1B2), 19.8% (F2B1), and 41.5% (F2B2) compared to the CK. The initial infiltration capacity of the F treatment was significantly lower than that of the CK treatment (*P* < 0.05). The possible reason was attributed to the direct mixing of a high proportion (100 g/kg) of FGDG with saline-alkali soil in the surface layer (0–20 cm). CaSO_4_ is moderately soluble in solution. When it is applied in solid form, complete dissolution is not possible, thereby blocking the soil pores and resulting in a significant decrease in the initial soil infiltration. The decrease in *K* of other treatments demonstrated that the application of FGDG and biochar promoted the inhibition of infiltration in the saline-alkali soil. The model fitting results were in agreement with the experimental results. In addition, the parameter *a″* for each treatment decreased by 90.1% (F), 62.6% (B), 69.9% (F1B1), 34.5% (F1B2), 23.7% (F2B1), and 47.5% (F2B2), respectively, in the USDA-NRCS model compared to CK, indicating a reduction in the water infiltration caused by the soil amendments. Moreover, the model-fitted results were consistent with the actual infiltration data.

In this study, the Kostiakov model demonstrated the best fit in describing the cumulative infiltration within the saline-alkaline soil. Few studies have investigated the effect of FGDG and biochar on soil water infiltration properties. Based on the prior research, the two-parameter models (Philip, Kostiakov, and USDA-NRCS) were less accurate than the three-parameter models (Mezencev and Horton) (Shukla et al., 2003; Dashtaki et al., 2009; Duan et al., 2011; Wang et al., 2017a). However, the Kostiakov and USDA-NRCS models performed better in this study. The probable reason is attributed to the application of FGDG and biochar with extensive pore structure and high specific surface area significantly altering the soil structure and total porosity (Zou et al., 2022).

### Effect of various experimental treatments on soil water content following infiltration

**Fig. 4:**
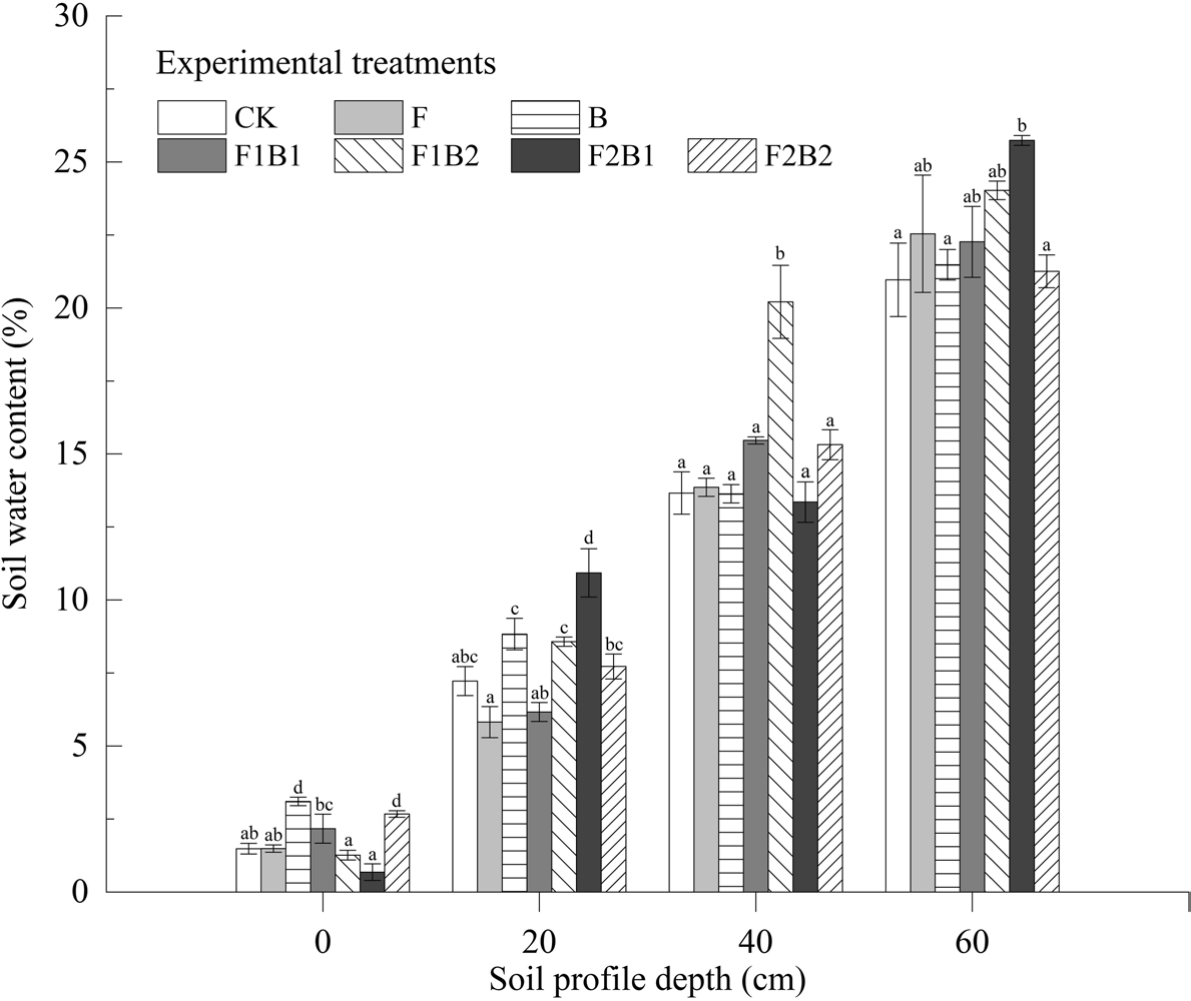
Effect of various experimental treatments on soil water content. Error bars represent the standard deviation. Mean values labeled with the same letter are not significantly different at *α* = 0.05

Soil water content following infiltration was measured to examine the effect of each experimental treatment on the water-holding capacity of the soil (Fig. 4). Soil water content increased with an increase in the depth of the soil layer. During infiltration, water permeated to the depth of the soil column. At the end of the infiltration process, water content was generally higher in the bottom layer than in the top layer of soil. The average water content in the 0–60-cm soil layer for all treatments followed the trend F1B2 > F2B1 > B > F2B2 > F1B1 > F > CK, i.e., 24.8%, 17.1%, 8.6%, 8.4%, 6.4%, and 0.9% higher than that of CK, respectively, indicating an increase in the water holding capacity of the soil by both single and combined treatments. The effect of combined treatments (F1B2 and F2B1) was more significant than that of single treatments. The soil water content in the F1B2 and F2B1 treatments varied significantly compared to CK (*P* < 0.05). Further, these combined treatments resulted in the highest water retention capability.

This study concluded that FGDG and biochar have the potential to significantly enhance the water-holding capacity of sandy soil, and the impact of the combined FGDG and biochar application is more significant than that of applying either treatment. The mixture of these two amendments had a greater synergistic effect on the structure of sand. FGDG mainly contributed Ca^2+^ ions to the soil, whereas biochar provided organic components needed for soil colloid formation and promoted the formation of stable soil aggregate structures (Koralegedara et al., 2019; Saifullah et al., 2018). The improvement in pore space of the soil decreases the macroporosity of the original sand, which, in turn, leads to a decrease in the soil water transport capacity. However, it also enhances the soil water retention capability (Al-Kayssi & Mustafa, 2016; Ibrahim et al., 2017; Spokas et al., 2014).

### Effect of various experimental treatments on total porosity of soil following infiltration

**Fig. 5:**
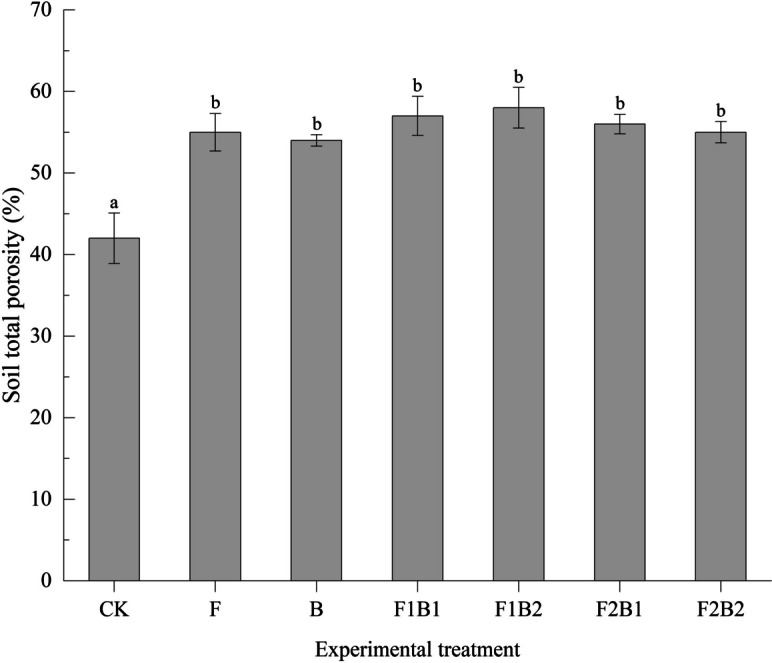
Effect of various experimental treatments on total porosity of surface soil (0–20 cm). Error bars represent the standard deviation. Mean values labeled with the same letter are not significantly different at *α* = 0.05

The influence of various experimental treatments on the total porosity of surface soil (0–20 cm) is given in Fig. 5. All treatments with amendments significantly increased total soil porosity compared to CK in the following order: F1B2 > F1B1 > F1B2 > F2B2 > F > B, i.e., an increase by 38.1%, 35.7%, 33.3%, 31%, 31%, and 28.6%, respectively. The combined treatments significantly improved the total soil porosity compared to the single treatments. Among the treatments, F1B2 had a more significant influence on total soil porosity.

The single or combined application of FGDG and biochar significantly increased the total porosity of the soil, which is in agreement with the previous studies (Blackwell et al., 1991; Chartres et al., 1985; Tirado-Corbala et al., 2019; Yu et al., 2014). The improvement in pore parameters could be attributed to the application of FGDG, which might have stimulated the formation of stable aggregates and inhibited their breakdown (Lebron et al., 2002; Tirado-Corbala et al., 2019; Yu et al., 2014).

Biochar has high porosity, which could alter the soil pore space upon its application (Rasa et al., 2018). The soil amended with biochar has enhanced porosity compared to the unamended soil (Githinji, 2014; Agegnehu et al., 2017; Esmaeelnejad et al., 2017). The pores within biochar particles as well as between biochar and soil particles increase the overall porosity of soil upon application of biochar (Agegnehu et al., 2017; Esmaeelnejad et al., 2017; Wu et al., 2022). Thus, the combined treatment has the potential to synergistically improve the physical structure of saline-alkali soil (sandy soil) in terms of the total soil porosity.

### Effect of various experimental treatments on soil pH following infiltration

**Fig. 6:**
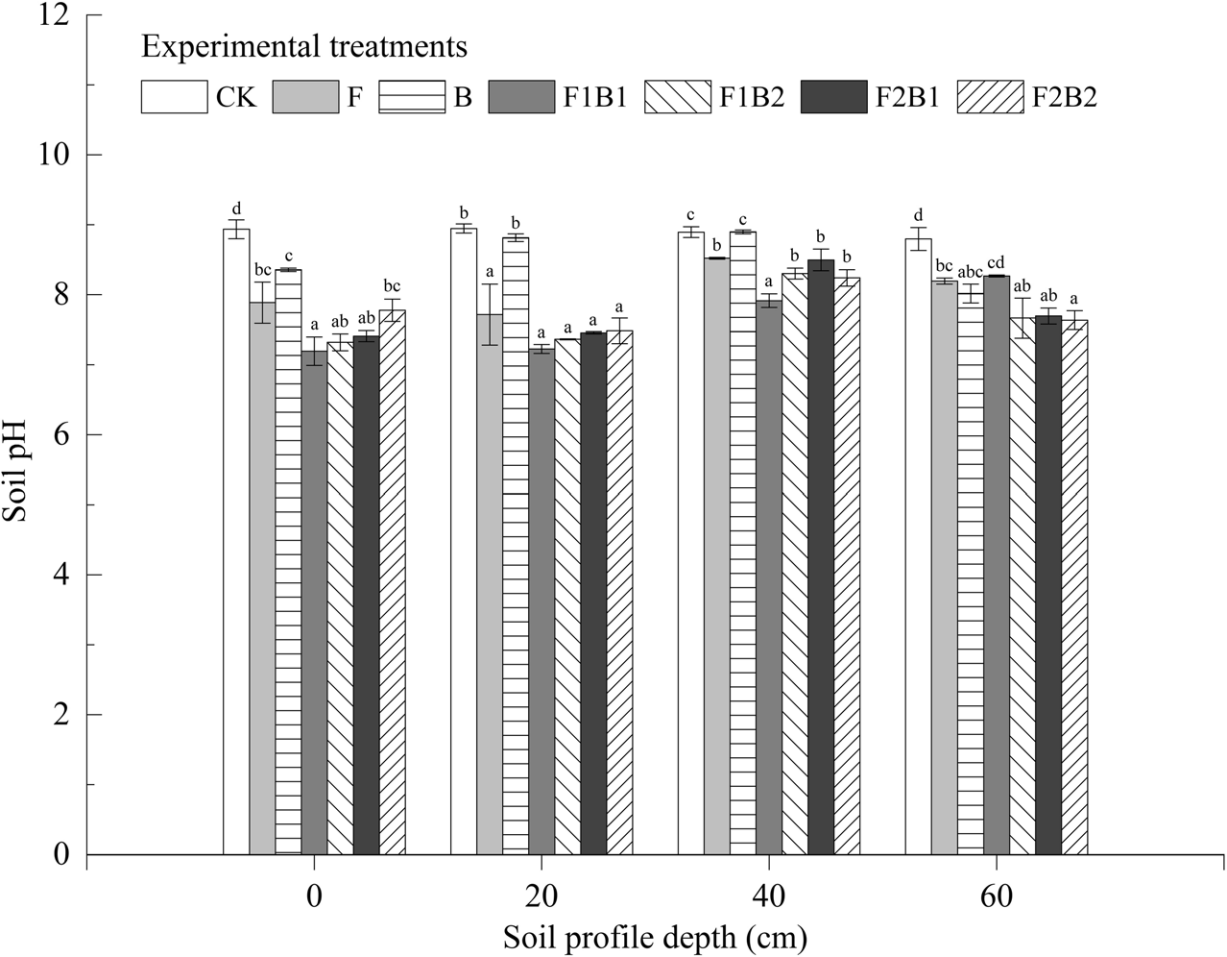
Effect of various experimental treatments on soil pH. Error bars represent the standard deviation. Mean values labeled with the same letter are not significantly different at *α* = 0.05

Figure 6 shows the results of soil pH measured at the end of infiltration. In general, the pH of the soil profile treated with amendments exhibits a pattern of initial increase followed by a decrease. A comparison of the mean pH values of the 0–60-cm soil layer for each treatment revealed that all treatments with amendments had lower pH compared to CK. The difference in pH was observed among treatments, in the order of increasing pH: F1B1 (7.65) < F1B2 (7.66) < F2B1 (7.76) < F2B2 (7.78) < F (8.08) < B (8.52) < CK (8.89). When compared with CK, the mean pH values of the 0–60-cm soil layer in other treatments decreased by 13.9% (F1B1), 13.8% (F1B2), 12.7% (F2B1), 12.5% (F2B2), 9.1% (F), and 4.2% (B). In addition, the mean pH of the soil from the surface layer (0–20 cm) of six treatments decreased by 19.4% (F1B1), 17.9% (F1B2), 16.9% (F2B1), 14.7% (F2B2), 12.8% (F), and 4% (B) compared to CK. A significant improvement in pH was observed in the surface soil layer than in the bottom soil layer. The B treatment had a slightly lower pH than that in CK; however, the difference was insignificant (*P* > 0.05). Therefore, the single or combined application of FGDG can significantly decrease the soil pH, and the combination of FGDG and biochar has significant influence than that of a single treatment. The combined treatments (F1B1 and F1B2) provided the best result among all treatments.

In this study, a single or combined application of FGDG led to a reduction in the soil pH, which is consistent with the previous studies (Goncalo et al., 2020; Zhang et al., 2020a, b; Wang et al., 2021; Morsy et al., 2022), and the mechanism is as follows (Koralegedara et al., 2019):

9 $$\mathrm{Na}_{2}\mathrm{CO}_{3}+\mathrm{CaSO}_{4}\rightarrow\mathrm{CaCO}_{3}\downarrow+\mathrm{Na}_{2}\mathrm{SO}_{4}$$

10 $$2\mathrm{Na}\mathrm{HCO}_{3}+\mathrm{CaSO}_{4}\rightarrow\mathrm{Ca}(\mathrm{HCO}_{3})_{2}\downarrow+\mathrm{Na}_{2}\mathrm{SO}_{4}$$

11 $$\mathrm{2Na^{+}(Soil\;colloid)+CaSO_{4}\rightarrow\;Ca^{2+}(Soild\;colloid)+Na_{2}SO_{4}}$$

Upon application of FGDG to the saline-alkali soil, the two reactions mentioned in Eqs. (9) and (10) occur in the soil solution, which resulted in the conversion of alkaline salts (Na_2_CO_3_ and NaHCO_3_) into neutral salts (Na_2_SO_4_ and CaCO_3_) followed by precipitation of these salts. As the reaction progressed, there was a decrease in the CO_3_^2−^ and HCO_3_^−^ contents as well as soil pH. In addition, exchangeable cations, such as Na^+^, K^+^, Ca^2+^, and Mg^2+^, were adsorbed on the surface of the saline-alkali soil colloid. FGDG provides a large concentration of Ca^2+^ ions, which react with exchangeable Na^+^ on the surface of a soil colloid. Subsequently, the remaining SO_4_^2−^, a strong acid radical, plays a vital role in neutralization (Koralegedara et al., 2019) and then lowers the soil pH.

However, an increase in the amount of FGDG does not necessarily improve the pH reduction. Improvement in pH of the soil treated with 10% FGDG alone was lower than the results of the other four combined treatments. This may be attributed to the excessive application of FGDG. The reactions in Eqs. (9), (10), and (11) continue to exert a positive influence. Na_2_SO_4_ accumulation in the soil solution was significantly high. There was an increase in the infiltration time in the soil treated with 10% FGDG, and Na^+^ ions were not released from the soil. Further, the reaction occurs in the opposite direction, leading to an increase in CO_3_^2−^ and HCO_3_^−^ and a gradual increase in soil pH. Therefore, there was no positive correlation between the pH reduction and the quantity of FGDG.

The alkalinity of biochar is the primary barrier to its application in saline-alkali soil. Numerous studies reported that the amendment of biochar or acidified biochar to saline-alkaline soil decreases its pH (Chaganti & Crohn, 2015; Khalifa & Yousef, 2015; Kim et al., 2016; Liu et al., 2017; Luo et al., 2017; Sun et al., 2017; Wu et al., 2014). However, a few studies have revealed that incorporating biochar into coastal saline soil increases the soil pH (Chen et al., 2008; Laird et al., 2010; Singh et al., 2022; Wang et al., 2017b; Yuan & Xu, 2011). The reduction in pH of saline-alkaline soil was not significantly influenced only by biochar amendment in this study. This may be attributed to the high pH of bamboo biochar and its alkaline effect on soil pH (Saifullah et al., 2018). The difference in pH between biochar and soil has affected the pH reduction (Liu & Zhang, 2012).

The effect of combined treatments on pH reduction was significant compared to the treatment with biochar alone. Firstly, the FGDG was involved in the chemical reaction (5). Secondly, the alkaline effect of FGDG was not affected by the addition of biochar in the combined treatment, but the synergistic effect of the two amendments improved the soil structure. As a result, the slow infiltration rate and water movement can provide sufficient time for the water-salt reactions in the soil. The increased water retention also provides the necessary aqueous conditions for the reaction. Thus, the combined application of FGDG and biochar has a significant impact on the reduction in the pH of saline-alkaline soil. However, it is necessary to manage the rate of application of both amendments.

### Effect of various experimental treatments on soil EC post infiltration

**Fig. 7:**
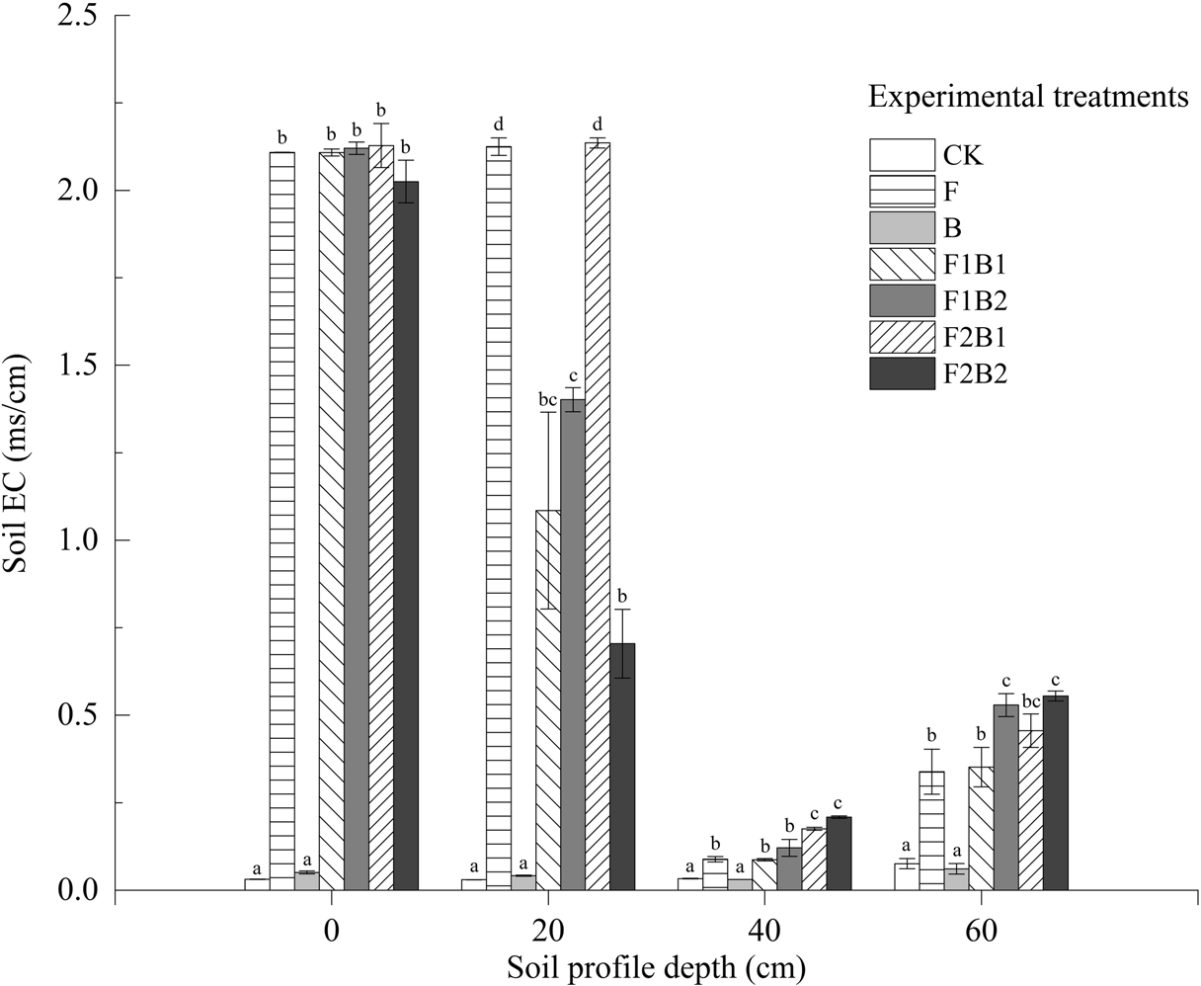
Effect of various experimental treatments on soil EC. Error bars represent the standard deviation. Mean values labeled with the same letter are not significantly different at *α* = 0.05

The EC of soil was measured at the end of infiltration, as shown in Fig. 7. In all treatments except for the B treatment, the EC of each soil layer increased significantly compared to CK. The surface soil layer exhibited extremely high EC, with the average EC value of the F treatment being 67.6 times higher than that of CK. Further, the EC of soil decreased significantly after 20 cm, with the lowest value being at 40 cm. The reason for this phenomenon can be attributed to the direct mixing of amendments in the surface soil layer, and the salt content of the soil treated with FGDG alone was extremely high. At the end of infiltration, a large amount of FGDG remained in the soil, which significantly increased the EC of the surface soil. The EC of the 40–60-cm soil layer increased with an increase in soil depth, with the EC of the 60-cm soil layer being significantly greater than that of the 40-cm soil layer. During infiltration, salts along with soil water moved to the bottom soil layer, and all treatments exhibited a certain desalination effect. The EC values at 60 cm followed the following order: F2B2 > F1B2 > F2B1 > F1B1 > F, and the highest EC value was 7.3 times higher than that of CK. A comparison of the EC values of the bottom soil layer of all treatments revealed that the EC values at the 60-cm layer were 4.03, 2.6, 4.37, 2.66, and 3.82 times higher than those at the 40-cm layer in the F1B1, F2B1, F1B2, F2B2, and F treatments, respectively. The desalination effect improved with an increase in the amount of FGDG at the same level of biochar application. However, when the FGDG amount reached 10%, the results were significantly less effective than those of the combined treatments. These results demonstrated that a positive desalination effect is not always produced with an increase in the amount of FGDG. In addition, the EC value of the soil with B treatment was close to that of CK, indicating no significant impact of biochar amendment alone on soil EC.

In this study, the EC of the surface soil amended with FGDG was significantly higher than that of CK. The EC decreased from 0 to 40 cm and increased from 40 to 60 cm, which is consistent with previous findings (Wang et al., 2021). The major reason can be attributed to the release of large amounts of Ca^2+^ and SO_4_^2−^, which may lead to an increase in the total salinity of soil (Qadir et al., 2002; Wang et al., 2012). The improvement in saline-alkali soil involves the exchange of Na^+^ with other ions and the discharge of these ions through irrigation. In this study, the desalting effect of 10% FGDG alone was not significant, and the infiltration rate of this treatment was very low, which is not conducive for the leaching of harmful ions such as Na^+^ and HCO_3_^−^ in the soil solution (Qadir et al., 2002). Yu et al. (2014) reported a similar finding that with an increase in the amount of FGDG, the marginal benefit of desalinating saline-alkali soil becomes increasingly limited. The application of FGDG releases a large amount of salt that contributes to the replacement of harmful Na^+^ with Ca^2+^ ions, enhances the soil aggregate structure, and increases the total porosity of the soil (Baligar et al., 2011; Tirado-Corbala et al., 2019; Yu et al., 2014). Further, FGDG converts toxic Na salts into Ca salts in the soil, which are beneficial for crop growth and yield (Wang & Yang, 2018). The effect of biochar alone on soil EC was not significant, which can be attributed to the coarse texture of saline-alkali soil and the low application rate of biochar (Singh et al., 2022) in this study. Therefore, FGDG combined with biochar can improve the desalination of saline-alkali soil.

### Comprehensive analysis of various treatments on soil infiltration and physicochemical properties

A comprehensive analysis of the effects of each treatment on soil infiltration and physicochemical properties demonstrated the substantial enhancement achieved by the combined application of FGDG and biochar in contrast to either treatment in isolation. There was no significant difference among the four combined treatments with various application ratios. The F1B2 treatment significantly improved the physicochemical properties of soil such as infiltration, total porosity, water-holding capacity, and pH. Under the synergistic action of FGDG and biochar, there was an improvement in the soil pore structure, an increase in the water-holding capacity, and a moderate decrease in the infiltration rate. The enhanced physical properties of soil provide sufficient time and the necessary aqueous environment for the occurrence of soil water-salt reaction, thereby significantly lowering the pH of saline-alkali soil. In addition, the enhanced water retention capacity can dilute the salt content in the soil, thereby reducing the negative impacts of hazardous salts on plant growth.

The method, proportion, and quantity of amendment were found to be the direct determinants of the enhancement effect. In this study, for example, the direct contact between the surface soil (0–20 cm) and amendments had a more significant influence on pH decrease than the bottom soil layer. For this reason, future studies on soil leaching are necessary to demonstrate the effect of amendments on water and salt migration in the saline-alkaline soil after multiple cycles of leaching and to further improve the desalination effect of saline-alkali soil in combination with field irrigation strategies.

## Conclusions

In this study, seven different treatment schemes with FGDG and biochar were designed and implemented. The infiltration process of newly reclaimed saline-alkali soil was investigated using soil column experiments. Five models were employed to simulate the infiltration process. The effects of FGDG and biochar on the infiltration characteristics and physicochemical properties of coastal saline-alkali soil were examined. The key findings are as follows:

1. When compared with CK, the infiltration capacity of saline-alkaline soil was reduced by the application of FGDG alone or in combination with biochar, and the addition of FGDG alone had the highest inhibition effect. The combined treatments demonstrated that the degree of inhibition of the infiltration process increased with the increase in the amount of FGDG. The low amount of biochar used in the combined treatments had minimal effect on infiltration characteristics. The Kostiakov model was best fitted for the experimental data of soil cumulative infiltration, and the power function can describe the dynamic change in the wetting fronts.
2. All treatments led to an increase in the water content and total porosity of saline-alkali soil, and the effect of combined application was more significant. The single or combined application of FGDG decreased the pH and increased the EC of saline-alkali soil, while a single application of biochar had a low impact on the pH and EC of saline-alkali soil.
3. The best improvement ratio among various amendments was achieved with the F1B2 combination, which includes 75 g/kg FGDG and 30 g/kg biochar.

## Data Availability

The authors confirmed that the data supporting the findings of this study are available within the article.
